# Supplementary material for: Federated learning for multi-omics: A performance evaluation in Parkinson’s disease
Source: Patterns (N Y). 2024 Mar 1;5(3):100945. doi: 10.1016/j.patter.2024.100945 (PMC10935499; doi:10.1016/j.patter.2024.100945)
Supplement: Document S1. Figures S1 and S2, Tables S1–S6 [file mmc1.pdf]

Patterns, Volume *5*

Supplemental information

Federated learning for multi-omics:

A performance evaluation in Parkinson's disease

Benjamin P. Danek, Mary B. Makarious, Anant Dadu, Dan Vitale, Paul Suhwan Lee, Andrew B. Singleton, Mike A. Nalls, Jimeng Sun, and Faraz Faghri

Supplementary Information

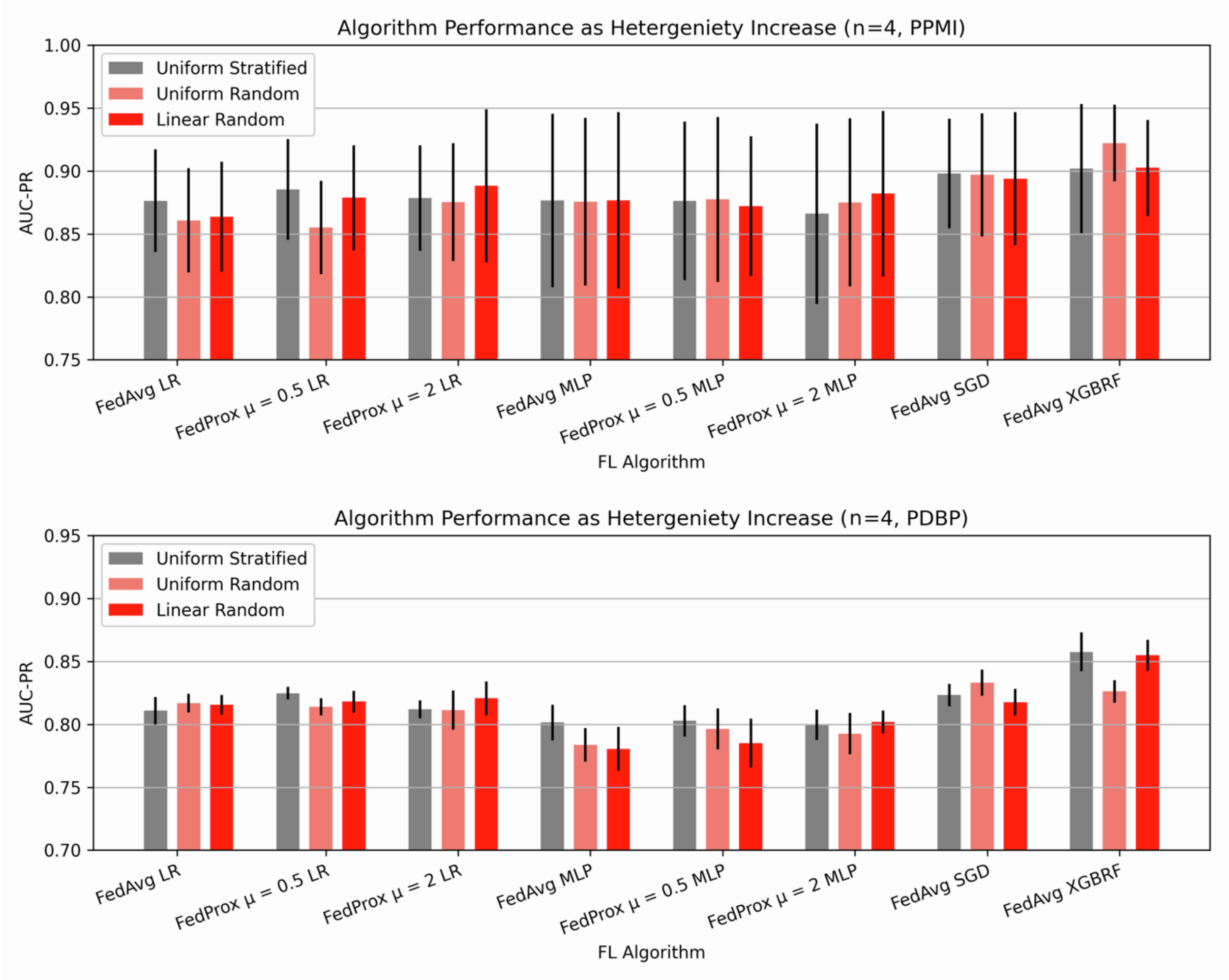

Supplementary Figure 1: The AUC-PR for a federation of 4 clients, for several split methods.

Uniform stratified sampling, representing the most homogenous data distribution method, while uniform random, and linear random represent increasingly heterogeneous client distributions. Presented data is mean score and standard deviation resulting from cross validation.

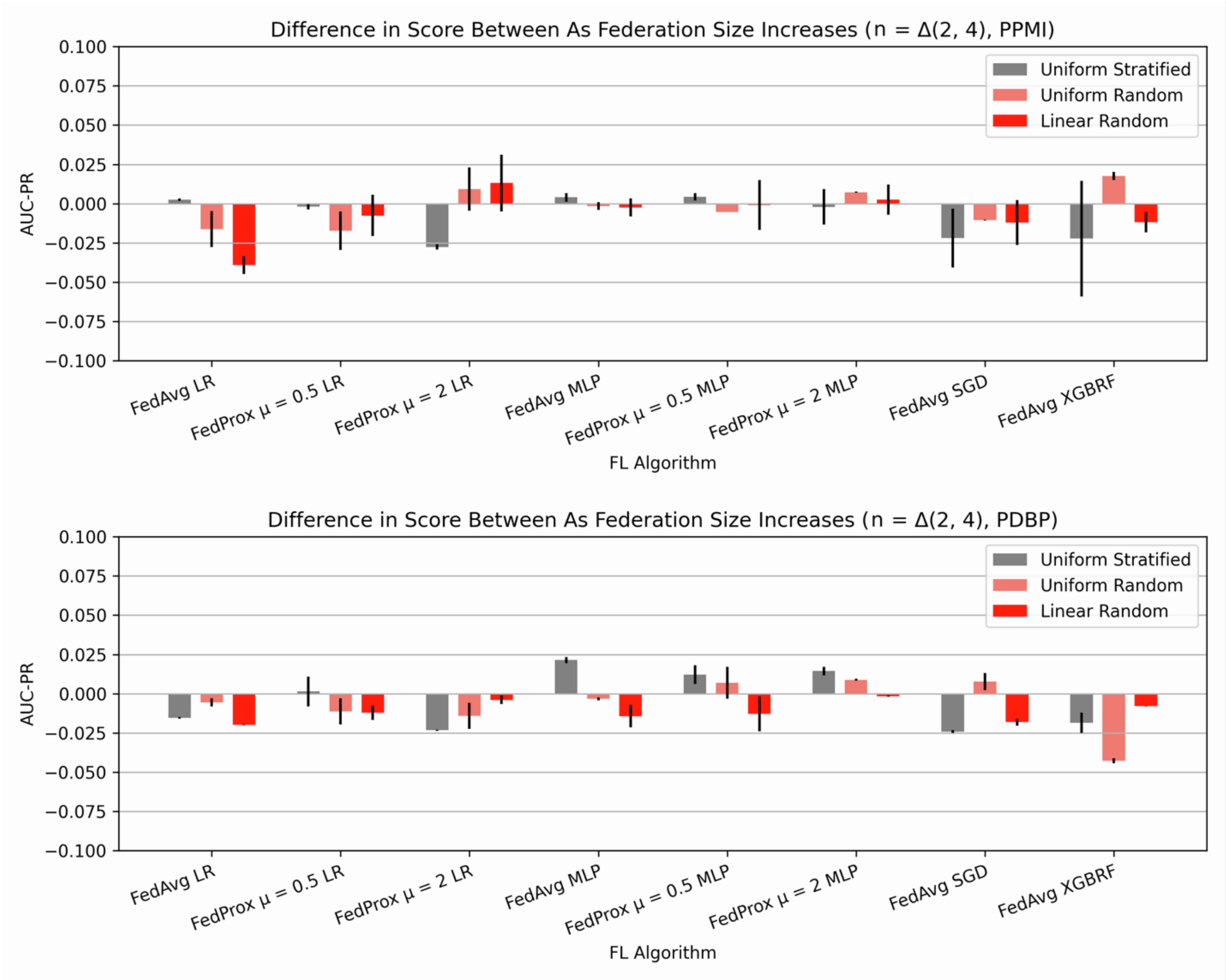

Supplementary Figure 2: The difference between AUC-PR scores between 2 and 4 clients, for several split methods.

Uniform stratified sampling, representing the most homogenous data distribution method, while uniform random, and linear random represent increasingly heterogeneous client distributions. Presented data is mean score and standard deviation resulting from cross validation.

Feature Name	Feature Source
Age	Clinico-demographic
Family History	Clinico-demographic
Male	Clinico-demographic
UPSIT	Clinico-demographic
Inferred Ashkenazi Jewish	Clinico-demographic
PRS90	Genetic
rs10182170	Genetic
rs10186643	Genetic
ENSG00000000938	Transcriptomic
ENSG00000001629	Transcriptomic
ENSG00000008394	Transcriptomic

Supplementary Table 1: The name and source of all clinic-demographic features, the first three genetic features, and the first three transcriptomic features. The comprehensive list of 674 features is available in the supplementary code repository.

Algorithm Name	Central Learner API	Federated Local Learner API	Federated Weight Aggregation Method	Federated Learning API
Logistic Regression	Scikit Learn	Scikit Learn	FedAvg, FedProx	Flower Framework
MLP Classifier	Scikit Learn	Scikit Learn	FedAvg, FedProx	Flower Framework
SGD	Scikit Learn	Scikit Learn	FedAvg	NVIDIA Flare
RF XGBoost Classifier	DMLC	DMLC	FedAvg	NVIDIA Flare

Supplementary Table 2: The description of frameworks used to implement central, and federated learning models.

	FedAvg LRClassifier	FedAvg MLPClassifier	FedAvg SGDClassifier	FedAvg XGBRFClassifier	FedProx μ = 0 LRClassifier	FedProx μ = 2 LRClassifier	FedProx μ = 0 MLPClassifier	FedProx μ = 2 MLPClassifier
**LogisticRegression**	greater*	greater*	greater	lesser	greater*	greater*	greater*	greater*
**MLPClassifier**	lesser*	lesser	lesser*	lesser*	lesser*	lesser*	lesser	lesser*
**SGDClassifier**	lesser*	lesser	lesser*	lesser*	lesser*	lesser*	lesser	lesser*
**XGBRFClassifier**	greater*	greater*	greater*	greater*	greater*	greater*	greater*	greater*

Supplementary Table 3: Method comparison table indicating statistical significance of the observed differences (greater, lesser) in performance measure (ROC-AUC) between models fit using central and federated methods on the external test set. Significance determined using DeLong's test, where an asterisk indicates statistical significance ($p < 0.05$).

	Case	Control	Total
Male	109	279	388
Female	62	147	209
Total	171	426	597

Supplementary Table 4: Value counts of PPMI cohort.

	Case	Control	Total
Male	185	451	636
Female	219	261	480
Total	404	712	1116

Supplementary Table 5: Value counts of PDBP cohort.

**Class Name**	**Algorithm Package**	**Package Version**
LogisticRegression	sklearn	1.3.0
RandomForestClassifier	sklearn	1.3.0
AdaBoostClassifier	sklearn	1.3.0
GradentBoostingClassifier	sklearn	1.3.0
SGD	sklearn	1.3.0
SVC	sklearn	1.3.0
MLPClassifier	sklearn	1.3.0
KNNClassifier	sklearn	1.3.0
LinearDiscriminantAnalysis	sklearn	1.3.0
BaggingClassifier	sklearn	1.3.0
XGBClassifier	xgboost	1.7.6
XGBRFClassifier	xgboost	1.7.6

Supplementary Table 6: The class names, algorithm packages, and package versions used to implement local learners in federated models, and central machine learning models.
